# Relationship between positive parenting and cyberbullying perpetration among adolescents: role of self-esteem and smartphone addiction

**DOI:** 10.3389/fpsyg.2023.1252424

**Published:** 2024-03-01

**Authors:** Ji Hye Kim, Hye Young Song, Gye Hyun Jung

**Affiliations:** ^1^Department of Nursing, Woosuk University, Wanju-Gun, Republic of Korea; ^2^Department of Nursing, Jeonbuk Science College, Jeongeup, Republic of Korea

**Keywords:** adolescents, cyberbullying, parenting, self-esteem, smartphone addiction

## Abstract

**Introduction:**

Adolescents may perpetrate cyberbullying in cyberspace, which they perceive as a medium of social communication, and cyberbullying perpetration is closely related to adolescent behavior, mental health, and development. This study aimed to examine the relationship of certain factors related to cyberbullying in adolescents using the framework of Jessor’s problem behavior theory.

**Methods:**

This cross-sectional study investigated the mediating effect of adolescent self-esteem and smartphone addiction in the relationship between positive parenting and adolescent cyberbullying perpetration. The secondary analysis used data from the 2018 Korean Children and Youth Panel Survey. The data on positive parenting, adolescent self-esteem, smartphone addiction, and perpetration of cyberbullying of 2,394 Korean adolescents (boys: 1,297, 54.2%) were analyzed. Hayes’ PROCESS macro program was used to confirm the mediating role.

**Results:**

The results revealed that 26.5% (*n* = 634) of adolescents had perpetrated cyberbullying. Positive parenting did not directly relate to cyberbullying perpetration. Adolescent self-esteem and smartphone addiction played a mediating role between positive parenting and adolescent cyberbullying.

**Discussion:**

Individual adolescent characteristics and parent/family system characteristics should be considered in policies aimed at preventing adolescent cyberbullying perpetration, preceded by the management of appropriate smartphone use. Improving young people’s self-esteem and teaching them how to use smartphones correctly can help prevent cyberbullying.

## 1 Introduction

Cyberspace is an online platform that creates and strengthens social relationships among young people. In cyberspace, adolescents experience various functional benefits, such as acquiring useful information or identifying social issues, feeling psychological satisfaction, and relieving stress (Kim, 2022). However, since adolescents perceive cyberspace as a medium for social communication, they may perpetrate cyberbullying, such as swearing, criticism, threats, and other forms of bullying (Chen et al., 2023). The perpetration rates of cyberbullying by South Korean youth increased from 22.8% in 2020 to 29.2% in 2021 (Korea Communications Commission. 2021, 2022), and its prevalence is believed to be increasing, representing a substitute for physical violence. Experiencing cyberbullying (i.e. being the victim) increases the risk of anxiety, depression, low self-esteem, emotional pain, substance use, and suicidal behavior in adolescents, requiring efforts to reduce this phenomenon (Katz et al., 2019). Cyberbullying perpetration is closely related to adolescent behavior, mental health, and development, and can be considered a serious psychiatric health problem (Zhu et al., 2021). To reduce these problems, efforts at home should be made first.

Jessor’s problem behavior theory (PBT) (Jessor and Jessor, 1977) explains the relationship between the perceived environment system, personality system, and behavior system in a socio-psychological framework, hypothesizing that the presence of one type of problem behavior in adolescents may increase the likelihood of developing another problem behavior (Vazsonyi et al., 2008; Zhu et al., 2021). The perceived environment consists of social influencing factors regarding the preferences and support of family and friends, and the personality system includes social cognitive variables related to values, beliefs, attitudes, and preferences for oneself and society. The behavioral system includes problem behaviors such as delinquent behavior, substance use, and early sexual activity that violate social and legal norms and tend to elicit some form of social control (Zhu et al., 2021).

According to the PBT theory, the adolescent’s family environment is associated with the adolescent’s problem behavior. Characteristics of the family, such as parenting attitudes and beliefs, were found to affect adolescent cyberbullying perpetration rates (Zurcher et al., 2018) Positive parenting is associated with parental warmth, support, participation, and open parent–child communication characteristics (Yu and Shek, 2021), and is defined by praise, encouragement, autonomy, and consistency (Seay et al., 2014). Positive parenting can bring beneficial changes in adolescents’ emotional stability, psychosocial development, and academic achievement (Seay et al., 2014; Kim and Kim, 2021).

Positive parenting requires the communication of constructive emotions as well as verbal and non-verbal expressions that confirm to adolescents that they are valued (Kim and Kim, 2021). Such parenting impacts adolescents’ emotional stability, psychosocial development, and academic achievements (Rosenberg, 1965). Positive interaction with parents can prevent exposure to the risk of cyberbullying perpetration and further increase mental health benefits for adolescents (Katz et al., 2019). Therefore, positive parenting may help prevent or reduce cyberbullying perpetration risk. Additionally, since positive interaction with parents can prevent exposure to the risk of cyberbullying perpetration and maintain the healthy mental health of adolescents (Katz et al., 2019), identifying how positive parenting can reduce or prevent the risk of cyberbullying perpetration is necessary.

Self-esteem refers to one’s self-evaluation of one’s social role, formed through love, acceptance, and positive interactions by reflecting on social and emotional experiences with significant others and their evaluations (Lei et al., 2020; Du et al., 2022). Positive parenting can change adolescents’ perception of their own self-esteem, lead to positive changes in friendship, academic, and school life satisfaction (Park, 2021; Reginasari et al., 2021; Du et al., 2022), and play an important role in controlling cyberbullying perpetration behavior (Mun and Choi, 2015; Kim and Kim, 2021). Furthermore, since positive parenting is an important factor in the formation of adolescents’ self-esteem (Mun and Choi, 2015), it is necessary to provide evidence for establishing a psychiatric health program that can improve adolescents’ self-esteem and reduce cyberbullying.

Moreover, adolescent smartphone addiction threatens adolescents’ psychological well-being, aggravates depression, promotes low self-esteem and fear of rejection, and impedes interpersonal relationship formation and development (Mun and Choi, 2015). Adolescents who are highly dependent on smartphones and active in cyberspace for a significant amount of time tend to witness or experience victimization by cyberbullying; exposure to such cyber environments leads to adolescents encountering threats such as cyberbullies (Chen et al., 2023). Positive parenting is an important factor that can change adolescent psychological and behavioral characteristics (Du et al., 2022), is linked to minimizing the risk of becoming addicted to smartphones, and can be an important variable in mediating the risk of becoming a perpetrator of cyberbullying (Katz et al., 2019; Garaigordobil and Navarro, 2022).

According to previous studies, the factors to prevent adolescents from partaking in cyberbullying were found to be affected by parental control style (Katz et al., 2019; Kim and Kim, 2021), empathy and emotional intelligence, parent–child relationship, school climate (Zhu et al., 2021), and parental support (Kim, 2022). The parenting attitudes of adolescents’ parents are more important than anything else because emotional control, problem-solving, and judgment can be less controlled in the adolescent (Kim and Kim, 2021). Therefore, positive parenting can serve as an important psychiatric health strategy to reduce cyberbullying perpetration behavior by improving adolescents’ self-esteem and reducing smartphone addiction.

Consequently, this study aimed to examine the relationship of certain factors related to cyberbullying in adolescents using the framework of Jessor’s problem behavior theory. A theoretical framework was created to evaluate their relationships. This study aimed to identify the associations among environmental and personal factors that contribute to the risk of cyberbullying perpetration in adolescents by examining the continuous mediating effect of self-esteem and smartphone addiction on the association between positive parenting style and cyberbullying rates. This study also aimed to present the theoretical foundation required to develop intervention strategies and programs that can help prevent cyberbullying perpetration.

The study hypotheses were as follows; Hypothesis 1: Positive parenting influence adolescent cyberbullying risk. Hypothesis 2: Self-esteem plays a mediating role in the relationship between positive parenting and adolescent cyberbullying perpetration. Hypothesis 3: Adolescent smartphone addiction plays a mediating role in the relationship between positive parenting and adolescent cyberbullying risk. Hypothesis 4: Adolescent self-esteem and smartphone addiction play a continuous mediating role in the relationship between positive parenting and adolescent cyberbullying perpetration risk.

## 2 Methods

### 2.1 Study design

This study was a secondary analysis using the Korean Children and Adolescent Panel Survey (KCYPS) and was a cross-sectional, descriptive study to verify the continuous mediating effect of adolescent self-esteem and smartphone addiction on the relationship between positive parenting and adolescent cyberbullying perpetration.

### 2.2 Participants

This study used data from the second year of the Korean Children and Youth Panel Survey 2018 (KCYPS 2018), a representative panel survey in South Korea. The data were obtained from the National Youth Policy Institute (2020). KCYPS 2018 was a nationally representative sample (National Youth Policy Institute, 2020). Multi-stage stratified cluster sampling was used to obtain a cohort of 2,500 fourth-grade elementary school students and first-grade middle school students, and 5,000 guardians from the original panel.

In the current study, the second survey of the KCYPS 2018 was used. Data were collected from August to November 2019. A trained interviewer visited households and conducted a Tablet-Assisted Personal Interview (TAPI) survey. The interviewer separated the original and guardian panels and conducted the survey using two independent questionnaires (one for students and one for guardians) simultaneously. Of 2,438 adolescents in 2019, 2,394 (boys: 1,297, 54.2%) were included in this study, excluding cases where a smartphone was not used or of there was a missing value (Table 1).

**Table 1:** Sociodemographic characteristics (*n* = 2,394).

	Categories	Total	Cyberbullying perpetration *n* = 634 (26.5%)	No cyberbullying perpetration *n* = 1,760 (73.5%)	*t* or χ^2^
		*N* (%) or M ± SD			
Sex	Boys	1,297 (54.2)	378 (59.6)	919 (52.2)	10.297**
	Girls	1,097 (45.8)	256 (40.4)	841 (47.8)	
Age		14.52 ± 0.33	14.52 (0.33)	14.53 (0.34)	−0.883
Family structure	Living with both parents	2,165 (90.4)	573.4 (89.3)	1,599 (90.9)	1.378
	Living with one parent	211 (8.8)	63 (9.9)	148 (8.4)	
	Not living with parents	18 (0.8)	5 (0.8)	13 (0.7)	
Perceived economic status	Low	310 (12.9)	85 (13.4)	225 (12.8)	3.006
	Average	1,862 (77.8)	501 (79.0)	1,361 (77.3)	
	High	222 (9.3)	48 (7.6)	174 (9.9)	
Perceived health status	Unhealthy	214 (8.9)	56 (8.8)	158 (9.0)	0.848
	Healthy	1,474 (61.6)	382 (60.3)	1,092 (62.0)	
	Very healthy	706 (29.5)	1.06 (0.17)	510 (29.0)	

^**^p < 0.01.

### 2.3 Materials

#### 2.3.1 Sociodemographic characteristics

The demographic characteristics of the participants included sex, age, household family structure, perceived economic level, and perceived health level. The family structure was classified into living with both parents, living with one parent, and others. Economic status was classified into “low/average/high” using parents’ responses to the item, “What is the economic status of your household?” Adolescents’ perceived health level was measured by their responses to the item, “How do you feel about your health compared to your peers?” as “not healthy at all,” “not very healthy,” “healthy,” and “very healthy.”

#### 2.3.2 Positive parenting

Positive parenting perceived by adolescents was measured by 12 items in “warmth,” “autonomy support,” and “structure “of the Parents as Social Context Questionnaire for Korean Adolescents (PSCQ-KA) scale developed by Skinner et al. (2005) and adapted into a Korean version by Kim and Lee (2017). Each item was scored on a 4-point scale (1 = “strongly disagree,” 4 = “strongly agree”). Cronbach’s α was 0.882, 0.836, and 0.766 for “warmth,” “autonomy support,” and “structure,” respectively, in the study by Kim and Lee (2017). Cronbach’s α was 0.907 in this study.

#### 2.3.3 Self-esteem

Self-esteem was measured using the Korean self-esteem scale (Jon, 1974). This scale consists of a total of 10 items. Each item was scored on a 5-point scale (1 = “strongly disagree,” 5 = “strongly agree”). A higher score indicated a higher level of self-esteem. Cronbach’s α was 0.930 in previous research (Jon, 1974) and 0.848 in this study.

#### 2.3.4 Smartphone addiction

Adolescents’ smartphone addiction was measured using the “Smartphone Addiction Proneness Scale” developed by Kim et al. (2012). This scale consisted of 15 items, including “My school grades drop due to excessive use of a smartphone,” “I have been criticized for frequently using a smartphone,” and “I feel restless and nervous without a smartphone.” Each item was scored on a 4-point scale (1 = “strongly disagree,” 4 = “strongly agree”), with a higher score indicating a higher level of smartphone addiction. Cronbach’s α was 0.814 in previous research (Kim et al., 2012) and 0.868 in this study.

#### 2.3.5 Cyberbullying perpetration

The perpetration of cyberbullying was measured using the cyberbullying perpetration scale developed by Lee et al. (2015). The scale comprises 15 items asking whether the respondent had committed 15 acts of bullying on a smartphone or on the Internet in the past year, including “I have personally sent swear words or harsh words to someone,” “I have stalked someone by sending words, texts, and images against his or her will,” and “I have repeatedly invited someone to an Internet chat room or prevented him or her from leaving against his or her will.” Each item was scored on a 6-point Likert scale, from 1 point for “never,” 2 points for “1–2 times a year,” 3 points for “once a month,” 4 points for “2–3 times a month,” 5 points for “once a week,” and 6 points for “several times a week,” with a higher score indicating a higher level of cyberbullying. By summing the values of each item, severe positive skewness was confirmed, and the values were converted into binary data (cyberbullying perpetration no = 0, yes = 1) for analysis. Cronbach’s α was 0.920 in this study.

### 2.4 Analysis methods

Analysis was performed using IBM SPSS 26.0 and PROCESS macro version 4.0. A frequency analysis examined the sociodemographic characteristics of the participants. The *t*-test and χ^2^ test were used to confirm the difference between the cyberbullying perpetration and non-cyberbullying perpetration groups. Pearson’s correlation analysis was conducted to identify the correlation between major variables. Hayes’s PROCESS macro program was used for analysis (model 6) to confirm the mediating role of self-esteem and smartphone addiction in the relationship between positive parenting and the perpetration of cyberbullying. Variables that showed a significant difference in cyberbullying perpetration were input as control variables. The significance of indirect pathways was confirmed using a bootstrapping technique. When checking the significance of the indirect path, samples were extracted and analyzed 5,000 times, with a 95% confidence interval.

### 2.5 Ethical considerations

This study was conducted in accordance with the guidelines of the Declaration of Helsinki and was approved by an institutional review board (approval number: WS-2022-32).

## 3 Results

### 3.1 Differences in cyberbullying perpetration according to sociodemographic characteristics

Overall, 26.5% (*n* = 634) of the participants had committed cyberbullying in the past year. Cyberbullying perpetration rates showed a significant difference according to sex (χ^2^ = 10.297, *p* < 0.001). Boys (54.2%) were more likely to commit cyberbullying than girls (45.8%). There was no significant difference in cyberbullying perpetration according to age (*t* = −0.883, *p* = 0.377), family structure (χ^2^ = 1.378, *p* = 0.502), perceived economic status (χ^2^ = 3.006, *p* = 0.222), and perceived health status (χ^2^ = 0.848, *p* = 0.655) (Table 1).

### 3.2 Correlation of main variables

Table 2 shows the correlation between independent and dependent variables. Adolescent cyberbullying perpetration showed a significant negative correlation with positive parenting (*r* = −0.065, *p* = 0.002) and self-esteem (*r* = −0.081, *p* < 0.001) and a significant positive correlation with adolescents’ smartphone addiction (*r* = 0.149, *p* < 0.001). Adolescents’ smartphone addiction showed a significant negative correlation with positive parenting (*r* = −0.296, *p* < 0.001) and self-esteem (*r* = −0.409, *p* < 0.001), and adolescents’ self-esteem showed a significant positive correlation with positive parenting (*r* = 0.510, *p* < 0.001).

**Table 2:** Descriptive statistics and correlations between main variables.

Variables	*M*	SD	1	2	3
1. Positive parenting	3.14	0.46	–		
2. Self-esteem	2.93	0.45	0.510^***^	–	
3. Smartphone addiction	2.13	0.46	−0.286^***^	−0.409^***^	–
4. Cyberbullying perpetration	0.26	0.44	−0.065^**^	−0.081^***^	0.149^***^

M, mean; SD, standard deviations; **p < 0.01, ***p < 0.001.

### 3.3 Mediating effect analysis

The PROCESS macro program was used to confirm the serial mediating effect of adolescent’s self-esteem and smartphone addiction in the relationship between positive parenting and adolescent cyberbullying perpetration. Among the sociodemographic characteristics, sex, which showed a significant relationship with cyberbullying perpetration, was input as a control variable.

**Figure 1:**
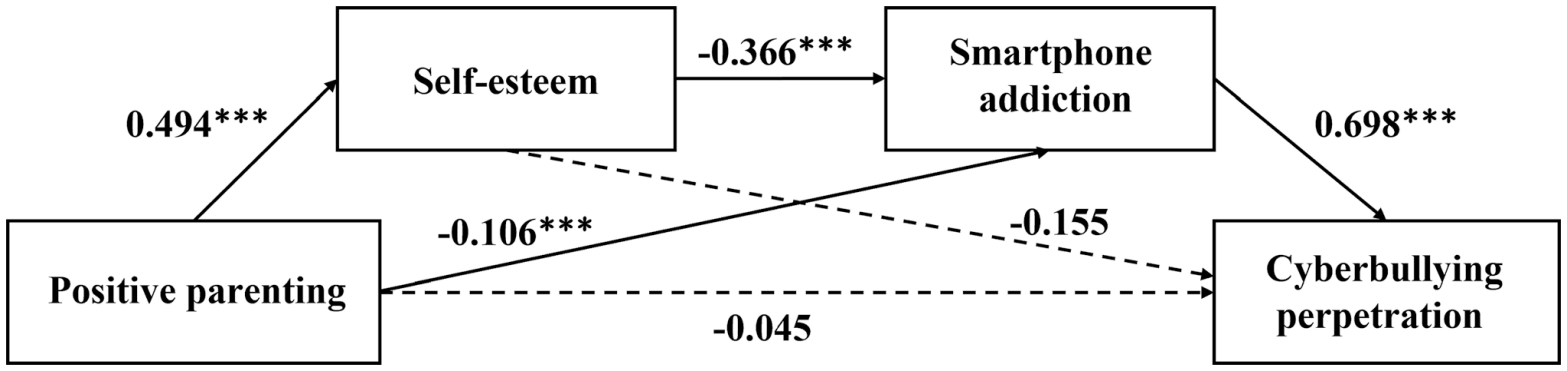
Serial multiple mediation with non-standard path coefficients. ***p < 0.001.

Table 3 and Figure 1 show the results of verifying the significance of the model paths. Positive parenting was significantly associated with adolescent self-esteem (*B* = 0.494, *t* = 29.383, *p* < 0.001), smartphone addiction (*B* = −0.106, *t* = −4.890, *p* < 0.001), but not with cyberbullying perpetration (*B* = −0.045, *Z* = −0.386, *p* = 0.700). Adolescent self-esteem was significantly associated with smartphone addiction (*B* = −0.366, *t* = −16.172, *p* < 0.001) but not with cyberbullying perpetration (*B* = −0.155, *Z* = −1.200, *p* = 0.230). Adolescent smartphone addiction was significantly associated with cyberbullying perpetration (*B* = 0.698, *Z* = 6.199, *p* < 0.001).

**Table 3:** Results of path analysis.

Path			*B*	se	*t* or *Z*	*p*	LLCI	ULCI
PP	→	SE	0.494	0.058	29.383	<0.001	0.461	0.527
PP	→	SA	−0.106	0.022	−4.890	<0.001	−0.149	−0.064
SE	→	SA	−0.366	0.023	−16.172	<0.001	−0.410	−0.321
PP	→	CP	−0.045	0.117	−0.386	0.700	−0.275	0.185
SE	→	CP	−0.155	0.130	−1.200	0.230	−0.409	0.098
SA	→	CP	0.698	0.113	6.199	<0.001	0.477	0.919

PP, Positive parenting; SE, Self-esteem; SA, Smartphone addiction; CP, Cyberbullying perpetration; CI, Confidence interval; LLCI, Lower limit CI; ULCI, Upper limit CI.

As a result of the bootstrapping, self-esteem and smartphone addiction fully mediated the relationship between positive parenting and cyberbullying perpetration, with a total indirect effect of −0.277 (CI [−0.410 to −0.146]). Specifically, the mediating effect was composed of indirect effects generated by three pathways: (1) positive parenting → self-esteem → cyberbullying perpetration (*B* = -0.077, CI [−0.210 to 0.057]); (2) positive parenting → smartphone addiction → cyberbullying perpetration (*B* = −0.074, CI [−0.119 to −0.037]); and (3) positive parenting → self-esteem → smartphone addiction → cyberbullying perpetration (*B*=−0.126, CI [-0.175 to −0.086]) (Table 4).

**Table 4:** Direct and indirect relations in the serial multiple mediation model.

	*B*	BootSE	BootLLCI	BootULCI
Direct effect	−0.045	0.117	−0.275	0.185
Total indirect effect	−0.277	0.067	−0.410	−0.146
1. PP → SE → CP	−0.077	0.067	−0.210	0.057
2. PP → SA → CP	−0.074	0.021	−0.119	−0.037
3. PP → SE → SA → CP	−0.126	0.022	−0.175	−0.086

PP, Positive parenting; SE, Self-esteem; SA, Smartphone addiction; CP, Cyberbullying perpetration; BootSE, Bootstrapping standard error.

As shown previously, cyberbullying perpetration rates are higher for boys than girls. Therefore, we analyzed the correlation of main variables between boys and girls separately. Girls’ cyberbullying perpetration showed a significant negative correlation with positive parenting (*r* = −0.090, *p* = 0.003) and self-esteem (*r* = −0.145, *p* < 0.001) and a significant positive correlation with smartphone addiction (*r* = 0.227, *p* < 0.001). On the other hand, only smartphone addiction was significantly correlated with boys’ cyberbullying perpetration (*r* = 0.093, *p* < 0.001). Additionally, when the mediating effect was checked by separating girls and boys, the results were the same as the overall results.

## 4 Discussion

This study aimed to evaluate the continuous mediating effect of self-esteem and smartphone addiction on the relationship between positive parenting and adolescent cyberbullying perpetration, based on Jessor’s PBT (Zhu et al., 2021). In this study, we showed the mediating effects of self-esteem and smartphone addiction on the relationship between positive parenting and adolescent cyberbullying perpetration.

As a result of a survey of cyberbullying perpetration based on general characteristics, there were more cyberbullying perpetration cases among men than women. This study (Polanin et al., 2022; Yang et al., 2022) found that male students reported more relational aggression and cyberbullying perpetration than female students. This type of cyber-aggression was found to be more noticeable when students are aware of their parents perceived hostility, neglect, and rejecting parenting attitudes.

From the results of the path analysis, Hypothesis 1: Positive parenting influence adolescent cyberbullying risk. Factors that can affect adolescent behavior are very diverse, ranging from genetic factors to socio-environmental factors. It is necessary to understand the social and physical environment surrounding an individual and their community to make meaningful conclusions about their behavior (Rosenberg, 1965). Therefore, it may not be possible to simply verify the relationship between parenting and adolescent cyberbullying perpetration without considering other influencing factors.

The path in which positive parenting mediated self-esteem and indirectly affected adolescent cyberbullying perpetration was not statistically significant, and Hypothesis 2 was rejected. These results are consistent with those of previous studies (Lereya et al., 2013; Seay et al., 2014), showing a positive correlation between positive parenting and adolescent self-esteem. These conflicting findings may indicate that self-esteem has an indirect effect on cyberbullying perpetration rates through its relationship with smartphone addiction, the preceding problem behavior, rather than being directly related to cyberbullying perpetration.

Hypothesis 3 was supported, as positive parenting had a significant indirect effect on cyberbullying perpetration by mediating smartphone addiction rates. While positive parenting plays an important role in the development of children’s psychological and behavioral control and parent–child communication in adolescence, it also improves the autonomy and control of adolescent behavior (Hofferth and Anderson, 2003). Moreover, positive parenting encourage moderation in the use of smartphones by adolescents (Augner and Hacker, 2012). Adolescents who are highly dependent on smartphones and active in the cyberspace for an extended period tend to witness or experience cyberbullying, which can increase the risk of perpetuating cyberbullying (Chen et al., 2023). Furthermore, disconnection from any social reality experienced in the cyberspace may aggravate the experience of cyberbullying perpetration.

Hypothesis 4 was supported, as positive parenting indirectly influenced cyberbullying perpetration rates through the sequential mediation of adolescent self-esteem and smartphone addiction. In adolescence, self-esteem tends to increase with positive experiences, driving values of self-esteem and a sense of self (Sallis et al., 2008). According to IPAR Theory (Rohner, 2016), parents’ accepting attitudes enable adolescents to value themselves and to evaluate themselves positively. Conversely, adolescents who perceive the rejection of parents have damaged self-awareness, leading to negative self-evaluation (Ramírez-Uclés et al., 2018). With low self-esteem, adolescents may feel alienated from their parents or friends and use smartphones to recover their self-esteem, leading to smartphone addiction (Andreassen et al., 2017). Further, adolescents with low self-esteem may commit delinquency to overcome a negative self-image; they may also struggle with impulse control (Kaplan, 1980). According to Jessor’s PBT (Zhu et al., 2021), taking part in one type of problem behavior increases the likelihood of taking part in another type of problem behavior since one problem behavior provides a socially organized opportunity to learn and practice other problem behaviors. Consequently, smartphone addiction may increase the risk of perpetuating cyberbullying. By testing Hypothesis 4, we have shown that positive parenting indirectly affected adolescent cyberbullying perpetration rates, mediated through self-esteem and smartphone addiction. Therefore, to help prevent adolescent cyberbullying, supporting positive parenting is required, as are interventions that consider these mediators.

The evidence presented in this study, which was based on Jessor’s PBT (Zurcher et al., 2018), suggests that the relationship between positive parenting and adolescent cyberbullying perpetration is complex and mediated by other factors. The analysis yielded statistically significant results, indicating the potential contribution of these factors on policy. Moreover, this evidence suggests a need for adolescent behavior experts to understand the continuity of problem behaviors that are embedded in PBT. In addition, factors such as adolescent self-esteem and smartphone addiction should be accounted for in intervention development.

This study had several limitations. First, this was a cross-sectional study, precluding meaningful discussions of causality. Longitudinal studies are required to evaluate the temporal and causal relationships among these variables. Second, this study included some sociodemographic characteristics of parents and adolescents; however, other factors may affect cyberbullying perpetration rates. Future studies should examine individual psychological factors as well as school- and community-related characteristics. This study collected data through a self-report questionnaire, and there is a possibility that the research participants did not report honestly, and there is a possibility that the data was underreported due to the tendency to answer in ways that are considered socially desirable, especially in relation to cyberbullying. Therefore, in future research, it would be beneficial to use or expand qualitative research methods such as observational research, in-depth interviews, and phenomenological research to compensate for the possibility of distortion of such information. Through qualitative research on the characteristics of the family system, there is a need to identify a positive parenting environment that can prevent cyberbullying among adolescents. Finally, the participants of this study came from East Asia. Positive parenting attitudes and cyberbullying perpetration by adolescents may be influenced by cultural factors such as moderation and filial piety (Wei and Liu, 2022), which were not applied in this study. Therefore, future studies should consider the mediating or moderating effects of cultural factors that may affect parents or adolescents.

## 5 Conclusion

This study aimed to evaluate the impact of positive parenting on adolescent cyberbullying perpetration rates, mediated by self-esteem and smartphone addiction. In this study, adolescent smartphone addiction and self-esteem were complete mediating factors in the relationship between positive parenting and adolescent cyberbullying perpetration rates; the relationship between the latter two variables was indirect. This evidence suggests that family system characteristics should be considered when designing interventions against cyberbullying perpetration, including smartphone addiction management.

## Data availability statement

Publicly available datasets were analyzed in this study. This data can be found here: 3. Korea Communications Commission. 2021. (2022). Cyberbullying survey report. Available online at: https://www.kcc.go.kr/user.do?mode=view&page=A02060400&dc=60400&dc=&boardId=1030&boardSeq=53091.

## Ethics statement

The studies involving humans were approved by Woosuk University institutional review board (approval number: WS-2022-32). The studies were conducted in accordance with the local legislation and institutional requirements. Written informed consent for participation in this study was provided by the participants’ legal guardians/next of kin. Written informed consent was obtained from the minor(s)’ legal guardian/next of kin for the publication of any potentially identifiable images or data included in this article.

## Author contributions

JK contributed to conception and design of the study, organized the database, and performed the statistical analysis. JK, HS, and GJ wrote the first draft of the manuscript and sections of the manuscript. All authors contributed to the article and approved the submitted version.
